# Spinning up the polymorphs of calcium carbonate

**DOI:** 10.1038/srep03616

**Published:** 2014-01-22

**Authors:** Ramiz A. Boulos, Fei Zhang, Edwin S. Tjandra, Adam D. Martin, Dino Spagnoli, Colin L. Raston

**Affiliations:** 1 Centre for NanoScale Science and Technology, School of Chemical and Physical Sciences, Flinders University, Bedford Park, SA 5042 Australia; 2 Department of Polymer Science and Engineering, School of Chemistry and Chemical Engineering, Shanghai Jiao Tong University, Shanghai 200240, China; 3 School of Chemistry and Biochemistry, The University of Western Australia, 35 Stirling Hwy, Crawley, WA 6009, Australia; 4 Department of Chemistry, The University of New South Wales, High St, Kensington, NSW 2052

## Abstract

Controlling the growth of the polymorphs of calcium carbonate is important in understanding the changing environmental conditions in the oceans. Aragonite is the main polymorph in the inner shells of marine organisms, and can be readily converted to calcite, which is the most stable polymorph of calcium carbonate. Both of these polymorphs are significantly more stable than vaterite, which is the other naturally occurring polymorph of calcium carbonate, and this is reflected in its limited distribution in nature. We have investigated the effect of high shear forces on the phase behaviour of calcium carbonate using a vortex fluidic device (VFD), with experimental parameters varied to explore calcium carbonate mineralisation. Variation of tilt angle, rotation speed and temperature allow for control over the size, shape and phase of the resulting calcium carbonate.

Carbon dioxide fixation from the atmosphere by marine organisms as large deposits of calcium carbonate is important in the carbon cycle. The rising concentration of carbon dioxide in the atmosphere, estimated to be 40% higher than preindustrial levels and higher than at any point in the last 800,000 years^1^, has led to a shift in the equilibrium of dissolved CO_2_ in seawater. This has lowered the pH of seawater and along with increasing temperatures, has greatly affected the optimum conditions for calcium carbonate (CaCO_3_) mineralisation^2^,^3^. Deposition of CaCO_3_ by marine organisms, in the form of aragonite in the nacreous layer or inner shell^4^,^5^,^6^, is therefore likely to be tested by the climate change^7^,^8^ in the Anthrapocene era^9^. Controlling the formation of CaCO_3_ polymorphs is important in understanding the mineralisation process with such changing environmental conditions, and for this purpose we used the recently developed, continuous flow processing, vortex fluidic device (VFD)^10^,^11^,^12^,^13^. While the conditions here are much more extreme and different to those likely to be encountered in the environment in the future, the results highlight the conditions required for selective preparation of different polymorphs of calcium carbonate. The vortex fluidic device (VFD), is capable of producing thin films in which high shear forces provide a mechanical energy which is effective in manipulating the growth of a range of diverse materials, including the synthesis of mesoporous silica^11^, decorating carbon nano-onions with palladium nanoparticles^12^, exfoliation of laminar material^10^, forming composite material with live algal cells and controlling organic reactions^13^.

Calcite is the most stable polymorph of CaCO_3_ and is the least soluble in water as opposed to vaterite, which is the least stable polymorph and most soluble in water. These stability differences arise from the way the calcium and carbonate ions are assembled in the extended solid-state structures, Fig. 1a^14^,^15^,^16^,^17^. Aragonite and calcite have similar structures with the inter-planar carbonate ions in a staggered arrangement relative to each other, minimising electrostatic repulsion, while in the less dense vaterite they are almost eclipsed relative to each other. The structures have been further explored in the present study using the recently developed Hirshfeld surface analysis^18^, in gaining further insight into the interplay of the ions in the solid state. Transformation from vaterite to aragonite and calcite is possible at 0–30°C and 60–80°C respectively^19^,^20^. In addition, aragonite is readily converted to calcite in nature at temperatures higher than 380°C^21^. The nucleation and growth of the three different polymorphs can be manipulated through the addition of crystal modifiers, including bio-polymers, inorganic salts, and macromolecules^22^,^23^. Developing protocols for gaining access to the different polymorphs of calcium carbonate is central to mimicking processes in nature, for example in generating synthetic aragonite, which has only recently been developed using polymer-mediated mineral growth, in addition to layer-by-layer deposition of a porous organic material^24^. Also important is gaining control over the interconversion of the different polymorphs of calcium carbonate where particular applications demand the presence of one phase of specific particle size and morphology (e.g. paper coating)^25^ while another application may require the *in situ* conversion of CaCO_3_ from one phase to another, such as in drug delivery^26^.

## Results

**Figure 1:**
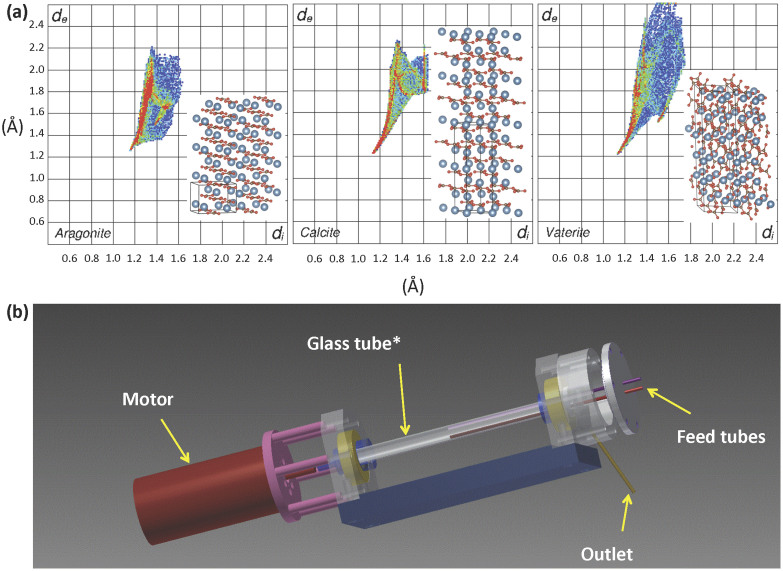
Structure analysis and processing device. (a) Hirshfeld surfaces of the calcium carbonate polymorphs, and (b) schematic of the vortex fluidic device (VFD). *Standard 10 mm NMR tube. 1(b) reproduced with permission from Bob Northeast.

The VFD used herein has a 10 mm tube with the capacity for high rotational speeds and variation in the tilt angle, θ, relative to the horizontal position, Fig. 1b. The latter can dramatically affect the shear forces present within the dynamic thin films^13^, which include solutions of Milli-Q water, seawater, and a mixture of ethanol and Milli-Q water, noting that ethanol is effective in CaCO_3_ polymorph control under conventional batch processing^27^. Milli-Q water spiked with NaCl matching the activity of salt in seawater and Milli-Q water spiked with 1% and 2% of Mg^2+^ also features in the study, noting that the presence of Mg^2+^ ions attenuates the growth of calcite with no effect on aragonite formation^28^,^29^. Aqueous solutions of NaHCO_3_ as the source of carbonate ions and aqueous solutions of CaCl_2_ as the source of calcium ions were fed into the VFD at a flow rate of 1 mL/min, in parallel with classical batch processing in a round bottom flask as control experiments. The effect of varying the ratios of HCO_3_^−^: Ca^2+^ on CaCO_3_ polymorph formation was investigated with relevance to the changing atmospheric and seawater concentration of CO_2_ (see experimental).

**Figure 2:**
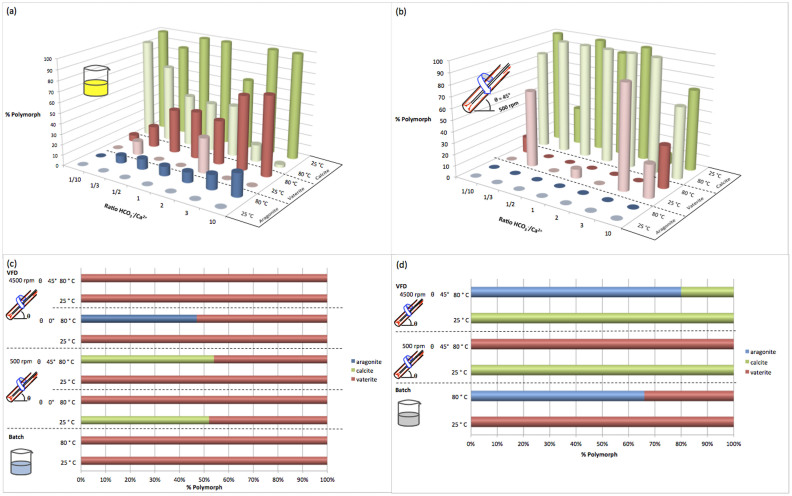
Effect of HCO_3_^−^/Ca^2+^ ratio, shear, and medium on polymorph synthesis. 3D plots for the ratio of HCO_3_^−^/Ca^2+^ at room temperature and 80°C for (a) batch processing and (b) VFD processing at 500 rpm, θ = 45°, against percent of the polymorph. 2D plots for CaCO_3_ polymorphs produced in seawater (c) and in 20% ethanol with Milli-Q water (d). VFD synthesis of CaCO_3_ at 4500 rpm, and 500 rpm at a tilt angle of θ = 0°, affording calcite exclusively. (VFD jet feed flow rates 1.0 mL/min). For clarity reasons, the order of temperature for calcite in (a) and (b) has been reversed.

For batch processing at room temperature, 71% of the samples produced calcite quantitatively, as established using X-ray diffraction (XRD) data, Fig. 2a. A 2:1 ratio of HCO_3_^−^ and Ca^2+^ results in a mixture of calcite (66.8%) and vaterite (33.2%), and decreasing or increasing the ratio of HCO_3_^−^: Ca^2+^ increased the percentage of calcite in the product at the expense of forming vaterite. For batch processing at 80°C there was little control in the formation of the CaCO_3_ polymorphs, with XRD data revealing a mixture of aragonite, calcite and vaterite in all but one sample. Nevertheless, as the ratio of HCO_3_^−^ to Ca^2+^ increases, the percentage of vaterite increases and to a lesser extent the percentage of aragonite increases, whereas the percentage of calcite decreases, Fig. 2a.

At room temperature, operating the VFD at 500 rpm at a tilt angle of 45° afforded a mixture of calcite and vaterite in 57% of the samples, while at 80°C the percentage of samples with both polymorphs dropped to 29%, Fig. 2b. Operating the VFD at 4500 rpm at a tilt angle of 45° gave no difference in the outcome for the continuous flow processing at room temperature and 80°C, with calcite being exclusively formed. The choice of 45° tilt relates to the angle that provides the greatest shear below 60° tilt. This has been judged by extensive optimisation of exfoliation of graphene and simple Diels Alder reactions^13^. Vaterite formation at 80°C was favoured at 1:10 and 10:1 ratios of HCO_3_^−^ to Ca^2+^. Reducing the angle of rotation of the VFD from 45° to 0° at 500 rpm also selectively afforded pure calcite for all ratios, irrespective of the temperature of the reaction.

We have shown that the VFD is effective in forming calcite or vaterite at high or low shear in aqueous systems with no evidence of the formation of aragonite despite its presence in nature in molluscan shells. Substituting Milli-Q water for seawater in the VFD and also in batch processing afforded vaterite quantitatively, Fig. 2c. Optimum conditions for obtaining vaterite in the VFD are low shear at 45° tilt angle (500 rpm), high shear at 45° tilt angle (4500 rpm) or 0° tilt angle, all at room temperature. Aragonite was produced in 45% yield at 80°C using the VFD at a rotation speed of 4500 rpm and 0° tilt angle. These results may reflect the presence of Mg^2+^ ions present in seawater inducing the formation of aragonite^30^. This was corroborated by XRD data for material prepared in the presence of Mg^2+^ ions, with 1% and 2% Mg^2+^ in Milli-Q water which resulted in 1% aragonite (99% calcite) in the VFD at operating at 500 rpm and 5000 rpm, at 45° tilt at room temperature. For the same conditions in the absence of added Mg^2+^, the product was exclusively calcite, and batch processing in the presence of 1% and 2% Mg^2+^ resulted in 1% aragonite (99% calcite) and 10% aragonite (90% calcite) respectively, in contrast to forming vaterite and calcite in the absence of Mg^2+^. The presence of carbonate and calcium ions in seawater made it inherently difficult to measure the concentration of the prepared NaHCO_3_ and CaCl_2_ solutions. Rather saturated solutions were prepared by the addition of excess CaCl_2_ and NaHCO_3_ to seawater followed by filtration. ICP-AES established that the seawater had 12500 ppm Na, 1237 ppm Mg, 891 ppm S, 389 ppm K, 400 ppm Ca, 8.9 ppm Sr and 1.35 ppm Si. (The Na and Si concentrations are higher than the global mean concentration in seawater of 10561 ppm and 0.03 ppm respectively, while that of Sr is lower than the average at 13 ppm.) Matching the activity of salt in seawater had no effect on aragonite formation with both batch and VFD processing resulting in 100% calcite.

Addition of ethanol induces aragonite formation^27^ and in the present study the effect of the presence of ethanol was studied for a 20% volume mixture of ethanol in Milli-Q water, using the VFD and also in batch mode as a control. For a stoichiometric ratio of HCO_3_^−^ to Ca^2+^ at 2:1, at room temperature, the VFD resulted in the formation of calcite regardless of the speed and tilt angle, Fig. 2d. In contrast, using the batch mode at room temperature, pure vaterite was obtained, while at 80°C a mixture of 64% aragonite and 34% calcite resulted. At higher temperatures and low shear stress using the VFD, vaterite was the exclusive product, while at a higher shear stress a mixture of aragonite and vaterite resulted. This is consistent with ethanol stabilising vaterite at higher temperatures by inhibiting its dissolution. Interestingly placing vaterite powder, which was prepared using the VFD operating in continuous flow mode, in water devoid of ethanol and now operating the VFD in the confined mode at a tilt angle of 45° and room temperature resulted in vaterite to calcite conversion. (The confined mode is for a finite volume of liquid processed in the VFD, where there can be intense shear at high speed in the absence of the viscous drag shear associated with continuous flow operation of the device^13^.) Replacing the water with hexane in the VFD operating under confined mode gave no such phase change and this is consistent with the phase change in Milli-Q water arising from the dissolution of vaterite and the precipitation of the calcium carbonate as calcite.

**Figure 3:**
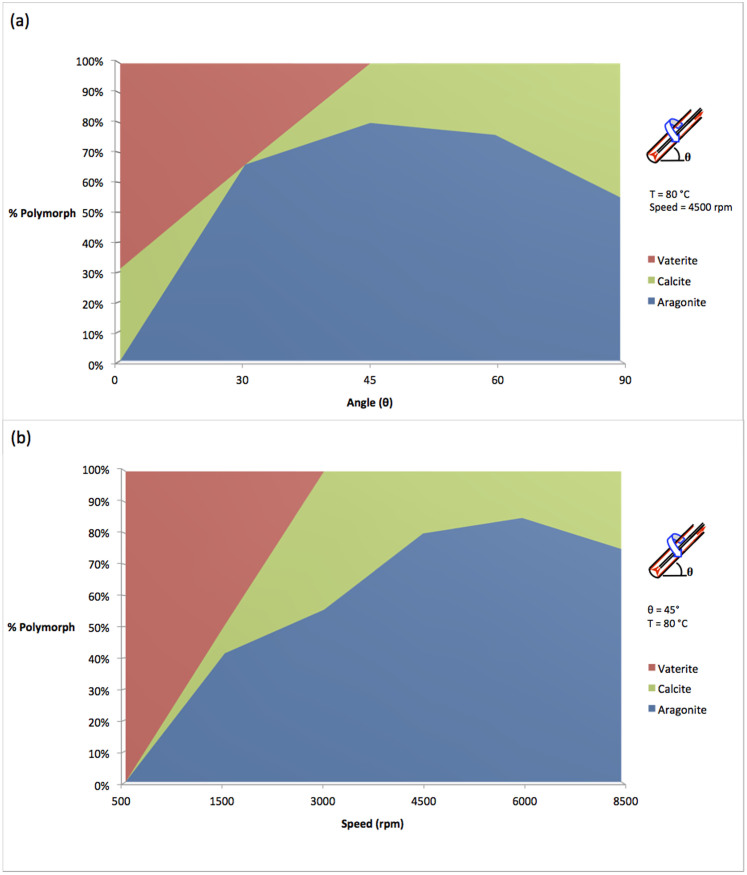
Rotational speed and tilt angle control on calcium carbonate polymorph formation. Varying the tilt angle (a) and speed (b) in 20% ethanol under continuous flow conditions, for flow rates of 1.0 mL/min.

The effect of tilt angle and speed on calcium carbonate synthesis in the VFD was studied for the ethanol/water system where reactant concentrations can be predetermined unlike in seawater, and where all three polymorphs can be produced, Fig. 2d. The effect of tilt angles at 4500 rpm and 80°C on the percentage of polymorph shows that for tilt angles below 45°, vaterite and aragonite dominate while at > 45°, calcite and aragonite dominate, Fig. 3b. Optimal conditions for aragonite synthesis occur at 45° with a yield of 80%. A plot of percentage of polymorph versus rotational speed at a tilt angle of 45° established that at 500 rpm vaterite is exclusively formed, and as the speed increases, the percentage of vaterite decreases to 0% at just under 4000 rpm, Fig. 3c. At 6000 rpm, aragonite and calcite are present in 80% and 20% respectively, a polymorph mixture that is not uncommon in molluscan shells^31^. Above 6000 rpm the percentage of aragonite decreases and that of calcite increases.

Hirshfeld surface analysis, as a recently developed tool for understanding the interplay of molecules and ions in the solid state^18^, highlights differences in the three polymorphs of CaCO_3_. This includes a variation in the percentage of O···Ca and O···O interactions in the Hirshfeld surfaces, at 63.8 and 20.4%, 63.9 and 25%, and 53.7 and 34.8%, respectively for vaterite, calcite and aragonite, Fig. 1a^18^. C···C interactions are absent in the Hirshfeld surfaces of vaterite and calcite, whereas there is a small component in that of aragonite (5.4%). More significantly, vaterite is distinctly different in having a significant component of C···O interactions between carbonate ions, making up 9.1% of the Hirshfeld surface for the polymorph, which appears as a spike in the bottom right hand side of the fingerprint plot^18^, Fig. 1a.

**Figure 4:**
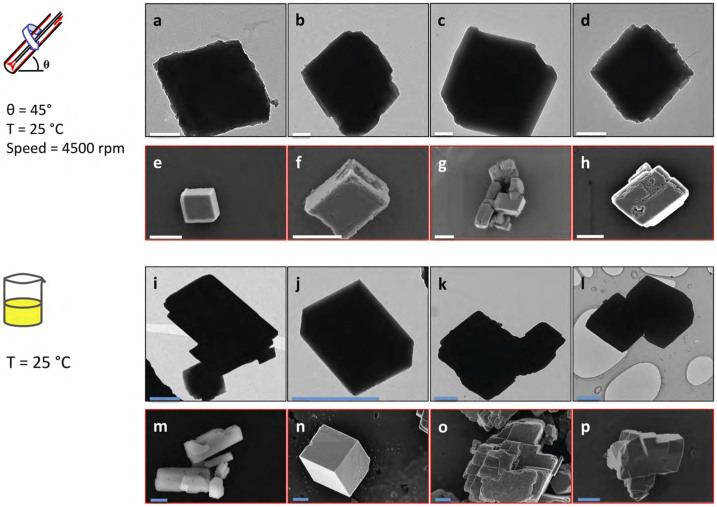
Control of size, shape and morphology. TEM and SEM images of samples showing morphologies of calcite obtained at room temperature using the VFD at 4500 rpm and 45° tilt angle (a–h), and using batch processing (i–p). Scale bar for images (a)–(d) is 200 nm and 1 μm for the other images.

As well as controlling the polymorphs of CaCO_3_, the use of the VFD is also effective in controlling the size and morphology of the particles. A comparison between the morphology using TEM and SEM for samples processed at 45° tilt angle and room temperature using the VFD establishes the ability to also control the shape and size of the calcite crystals, with an average size of approximately 1 μm, Fig. 4a–h. This is in contrast to little control in shape and size for calcite crystals produced using batch processing, Fig. 4i–p.

## Discussion

The results establish that at high temperature and high shear (4500 rpm) in Milli-Q water in the VFD results in the selective formation of the most stable polymorph, namely calcite. In contrast, operating the VFD at room temperature and low shear (500 rpm) favours exclusively vaterite, and this is consistent with previous experiments where CaCO_3_ produced in a spinning disc processor (SDP) at 500 rpm consists of the vaterite polymorph^32^. (The SDP relates to the VFD in providing a dynamic thin film, but on a disc rather than in a tube.) The high shear forces in the VFD resulting in the formation of calcite are akin to the mechanical energy associated with milling which is capable of inducing a vaterite to calcite transformation^33^.

Replacing the Milli-Q water with seawater in the VFD, however, results in the quantitative formation of vaterite at high shear (4500 rpm), at room temperature and 80°C. Presumably this arises from the high ionic activity of metal ions and carbonate ions in seawater preferentially precipitating CaCO_3_ and circumventing the dissolution of vaterite, Eq. 1. 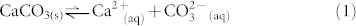
The ability to generate a mixture of aragonite and calcite at high shear (4500 rpm) and in 20% ethanol in the VFD, highlights the delicate balance between the these polymorphs, which is consistent with a relatively small energy difference between their formation energies^34^,^35^. This relates to similar crystal structures, which are distinctly different to that of vaterite, and this is further highlighted in the Hirshfeld surface analysis.

We have demonstrated the differential formation of crystalline CaCO_3_ polymorphs under shear using a vortex fluidic device (VFD), unlike in batch processing. The ratio of Ca^2+^ to HCO_3_^−^ is important in controlling the polymorph(s) using batch processing while using the VFD, calcite is obtained selectively at high shear, regardless of the tilt angle and temperature. Selective formation of vaterite using seawater under batch conditions occurs at room temperature and 80°C, and in the VFD at 45° and high shear. In the VFD up to 45% aragonite is accessible in seawater and up to 80% (and 20% calcite) in a mixture of ethanol and water, which is similar to the ratio of the polymorph in molluscan shells. Overall, the variation in shear and temperature in controlling the polymorphs of calcium carbonate has implications in understanding how marine organisms create their shells as conditions in the oceans change. The ability to control the size, shape and morphology of polymorphs of calcium carbonate is also significant, for a range of applications, from paper coatings to catalysis, to drug delivery, to templates for carbonaceous materials. Moreover, such control establishes a precedent for using the device to control the crystallisation outcome of a raft of materials.

## Methods

The VFD consists of a 10 mm NMR tube as the reaction chamber with reactants fed into the chamber with controlled flow rates using a peristaltic pump, Fig. 1b. The tilt angle of the VFD can be varied from 0° to 90° and the rotational speed can be varied from 500 rpm to 7000 rpm. A heat jacket equipped with a heat gun can be used to operate reactions above room temperature.

The CaCO_3_ was prepared by the reaction of sodium hydrogen carbonate (NaHCO_3_) and calcium chloride (CaCl_2_) to give calcium hydrogen carbonate (Eq. 2) which then decomposes to CaCO_3_, H_2_O and CO_2_ (Eq. 3). NaHCO_3_ was purchased from Optigen Scientific Pty. Ltd. and was of Optigrade™, and CaCl_2_ was purchased from Chem-Supply Pty. Ltd. and was of laboratory reagent quality. 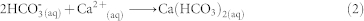


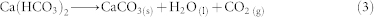
Stock solutions of NaHCO_3_ and CaCl_2_ were made in Milli-Q water to give a final concentration of 1 M. The reactants were prepared in 1:1, 1:2, 2:1, 1:3, 3:1, 1:10 and 10:1 ratios of HCO_3_^−^ to Ca^2+^. In the batch process, 15 mL of CaCl_2_ solution with the target concentration was added drop wise to 15 mL of NaHCO_3_ solution with the target concentration in a 50 mL conical flask. For the VFD under continuous flow, Fig. 1b, solutions of NaHCO_3_ and CaCl_2_ with the target concentrations were fed into the VFD at a flow rate of 1 mL/min using a peristaltic pump with the VFD set to the desired angle and at the desired temperature. The CaCO_3_ obtained was filtered, washed with Milli-Q water and left to dry at room temperature.

**Full methods** and any associated references are available in the online version of the paper at www.nature.com/nature.

## Author Contributions

R.A.B. and F.Z. carried out fluid flow experiments, R.A.B., F.Z. and E.S.T. carried out characterisation, A.D.M. carried out the Hirshfeld surface analysis, R.A.B., D.S. and C.L.R. designed the experiments and wrote the paper, and C.L.R. designed the microfluidic platform and coordinated the research.

## Supplementary Material

Supplementary Information Supplementary Information
