# Supplementary material for: Spinning up the polymorphs of calcium carbonate
Source: Sci Rep. 2014 Jan 22;4:3616. doi: 10.1038/srep03616 (PMC3898216; doi:10.1038/srep03616)
Supplement: Supplementary Information [file srep03616-s1.docx]

**Supplementary Information**

**Spinning up the polymorphs of calcium carbonate**

*Ramiz A. Boulos,^1^ Fei Zhang,^1,2^ Dino Spagnoli,^3^ Adam D. Martin,^4^ Edwin S. Tjandra,^3^ and Colin L. Raston^*1^*

1 Centre for NanoScale Science and Technology, School of Chemical and Physical Sciences, Flinders University, Bedford Park, SA 5042 Australia.

colin.raston@flinders.edu.au

2 Department of Polymer Science and Engineering, School of Chemistry and Chemical Engineering, Shanghai Jiao Tong University, Shanghai 200240, China

3 School of Chemistry and Biochemistry, The University of Western Australia, 35 Stirling Hwy, Crawley, W.A. 6009, Australia.

4 Department of Chemistry, The University of New South Wales, High St, Kensington, NSW 2052

**Methods**

TEM images were recorded using a 3000F JEOL instrument operating at 300 KeV. X-ray diffraction (XRD) was carried out on a Empyrean facility using a fine-focus copper tube X-ray source. Experiments were conducted at an accelerating voltage of 40 kV and a current of 40 mA with the angle of incidence and reflection of the incident ray ranging from 20° to 80°. HighScore software was used for visualizing and analysing the output files. SEM measurements were recorded using a Zeiss 1555 VPFESEM instrument operating at 15 KeV. The sample was dispersed in water and left to dry on a carbon tape mounted on a stub. The powder XRD spectra of samples produced in the presence of ethanol and in the presence of NaCl were collected on a Bruker D4 Endeavour Powder X-ray diffractometer at Adelaide University. A Co Kα radiation source was used and data was collected by scanning 2theta from 20° to 80° in 0.02 degree increments. Topaz was used for data processing of the acquired spectra.

Hirshfeld surface analysis revealed significant differences in the interactions between the ions in the crystal structures of aragonite, calcite and vaterite. Aragonite stacks in a staggered manner down the *c* axis, whereas calcite packs in an eclipsed manner, and in vaterite the carbonate groups are offset, resulting in neither a staggered or eclipsed packing arrangement. In all cases the fingerprint plots of the carbonate molecules appear “skewed” away from the d_i_ = d_e_ diagonal and this is due to the difference in atomic radii between the calcium and smaller oxygen and carbon atoms. Interestingly, the nature of the O∙∙∙O interactions differ for each polymorph. In vaterite, the interactions are due to longer interactions with adjacent carbonates, although as vaterite does not have a layered structure these interactions are more difficult to quantify. In aragonite the O∙∙∙O interactions are due to the orientation of one oxygen towards an adjacent oxygen from the same layer (i.e. in-plane interaction), however in calcite the O∙∙∙O interactions are solely due to interactions between oxygens in neighbouring layers (i.e. out-of-plane interactions). This is illustrated in Fig. 1a. This corresponds well with the staggered and eclipsed arrangements of carbonate moieties in aragonite and calcite respectively.

Hirshfeld surface analysis was performed using CrystalExplorer 2.1^2^, and VESTA was used for the structure presentation.^1^

Elemental composition of seawater was determined using a Thermo iCAP 6500 Inductively Coupled Plasma-Atomic Emission Spectrophotometer (ICP-AES) (Thermo Fisher Scientific, Australia). Seawater was collected in a PVC container and filtered through a 2.5 μm pore size (Whatman 5) to remove any suspended material. The sample was stored in a glass vial for transportation to the laboratory prior to analysis.  Samples were then diluted 40 x using 2% v/v quartz sub-boiling redistilled nitric acid (QCS GmbH, Germany), using a Hamilton Labs Auto diluter (Hamilton Company, USA). Element concentrations were determined relative to a series of multi-element standards (0, 1, 2, 5 and 10 μg.mL^-1^ in 5% v/v quartz sub-boiling redistilled nitric acid), prepared by serial dilution of Accutrace Reference Standard MES-04-5 (AccuStandard Inc., USA).

1. Momma, K & Izumi, F. VESTA 3 for three-dimensional visualization of crystal, volumetric and morphology data. *J. Appl. Crystallogr.* **44**, 1272-1276 (2011).

2. Wolff, S. K., Grimwood, D. J., McKinnon, J. J., Jayatilaka, D. & Spackman, M. A. *CrystalExplorer 2.1*, The University of Western Australia, 2008.
